# Pan-cancer immune and stromal deconvolution predicts clinical outcomes and mutation profiles

**DOI:** 10.1038/s41598-025-09075-y

**Published:** 2025-07-04

**Authors:** Bhavneet Bhinder, Verena Friedl, Sunantha Sethuraman, Davide Risso, Kami E. Chiotti, R. Jay Mashl, Kyle P. Ellrott, Jordan A. Lee, Christopher K. Wong, Kofi Gyan, Aditya Deshpande, Marcin Imielinski, Rohan Bareja, Josh Stuart, Myron Peto, Katherine A. Hoadley, Alexander J. Lazar, Andrew D. Cherniack, Jingchun Zhu, Shaolong Cao, Mark Rubin, Wenyi Wang, Oliver F. Bathe, Nicolas Robine, Li Ding, Peter W. Laird, Wanding Zhou, Hui Shen, Vésteinn Thorsson, Jen Jen Yeh, Matthew H. Bailey, Daniel Cui Zhou, Xianlu L. Peng, Mary Goldman, Yongsheng Li, Anil Korkut, Nidhi Sahni, D. Neil Hayes, Michael K. A. Mensah, Ina Felau, Anab Kemal, Samantha Caesar-Johnson, John A. Demchok, Liming Yang, Martin L. Ferguson, Roy Tarnuzzer, Zhining Wang, Jean C. Zenklusen, The Cancer Genome Atlas Analysis Network, Adam Margolin, Alana Weinstein, Andrea Sboner, Andrew Blair, Angela Brooks, Benjamin Berman, Benjamin Raphael, Brian Craft, Dante Bortone, David Heimain, David Gibbs, David Haan, Doron Betel, Duncan McColl, Emek Demir, Faeze Brahman, Farshad Farshidfar, Gaddy Getz, Galen Gao, Gordon Robertson, Huy Dinh, Hyo Young Choi, Ilya Shmulevich, Ioannis Anastopoulous, Jasmine Yang, Jeff Damrauer, Ken Chen, Lauren Sanders, Lee Cooper, Liang-Bo Wang, Matt Reyna, Mike Noble, Mohammed El-Kebir, Molly Zhang, Nicole Yeager, Paul Little, Richard Moffitt, Sam Meier, Teresa Swatloski, Theo Knijnenburg, Thomas Matthew, Vicky Chen, Vlado Uzunangelov, Xinghua Lu, Yige Wu, Yulia Newton, Zeya Wang, Paul Spellman, Olivier Elemento

**Affiliations:** 1 Englander Institute for Precision Medicine, Weill Cornell Medicine, New York, NY 10021 USA; 2 Institute for Computational Biomedicine, Weill Cornell Medicine, New York, NY 10021 USA; 3 Biomolecular Engineering Department, School of Engineering, University of California, Santa Cruz, Santa Cruz, CA 95064 USA; 4 UC Santa Cruz Genomics Institute, Santa Cruz, CA 95064 USA; 5 Division of Oncology, Department of Medicine, Washington University in St. Louis, 4515 McKinley Dr, St. Louis, MO 63110 USA; 6 Department of Statistical Sciences, University of Padova, Via C. Battisti 241, 35121 Padua, Italy; 7 Department of Molecular and Medical Genetics, Oregon Health & Science University, 3181 SW Sam Jackson Park Rd, Portland, OR 97239 USA; 8 McDonnell Genome Institute, 4444 Forest Park Ave Washington University, St. Louis, MO 63108 USA; 9 Department of BioMedical Engineering, Oregon Health & Science University, 3181 SW Sam Jackson Park Rd, Portland, OR 97239 USA; 10 Lineberger Comprehensive Cancer Center, Department of Genetics, University of North Carolina at Chapel Hill, Chapel Hill, NC 27599 USA; 11 Departments of Pathology, Genomics Medicine, Dermatology, & Translational Molecular Pathology, The University of Texas MD Anderson Cancer Center, 1515 Holcombe Blvd-Unit 85, Houston, TX 77030 USA; 12 Broad Institute of Harvard and MIT, Cambridge, MA 02142 USA; 13 Department of Medical Oncology, Dana-Farber Cancer Institute, Boston, MA 02215 USA; 14 Department of Bioinformatics and Computational Biology, The University of Texas MD Anderson Cancer Center, 1400 Pressler Street, Houston, TX 77030 USA; 15 Department for BioMedical Research, University of Bern, Bern, 3012 Switzerland; 16 Division of Surgical Oncology, Tom Baker Cancer Centre, 1331 - 29Th St NW, Calgary, AB T2N 4N2 Canada; 17 Departments of Surgery and Oncology, Cumming School of Medicine, University of Calgary, 3280 Hospital Drive NW, Calgary, AB T2N 4Z6 Canada; 18 New York Genome Center, Computational Biology, 101 Avenue of the Americas, New York, NY 10013 USA; 19 Van Andel Institute, 333 Bostwick AVE NE, Grand Rapids, MI 49503 USA; 20 Institute for Systems Biology, 401 Terry Avenue N, Seattle, WA 98109 USA; 21 Department of Pharmacology, Lineberger Comprehensive Cancer Center, University of North Carolina, Chapel Hill, NC 27599 USA; 22 Department of Systems Biology, The University of Texas MD Anderson Cancer Center, Houston, TX 77030 USA; 23 Department of Epigenetics and Molecular Carcinogenesis, The University of Texas MD Anderson Cancer Center, 1808 Park Rd 1C, Smithville, TX 78957 USA; 24 University of Tennessee Health Sciences Center, Memphis, TN 38103 USA; 25 National Cancer Institute, 31 Center Dr, 3A20, Bethesda, MD 20892 USA; 26 Department of Oncology and Arnie Charbonneau Cancer Institute, Cumming School of Medicine, University of Calgary, Alberta, T2N 4N1 CA Canada; 27 Emory University, Atlanta, GA 30322 USA; 28 Princeton University, Princeton, NJ 08540 USA; 29 The University of North Carolina, Chapel Hill, NC 27599 USA; 30 University of California Santa Cruz, Santa Cruz, CA 95064 USA; 31 University of Pittsburgh, Pittsburgh, PA 15206-3701 USA; 32 UT-MD Anderson Cancer Center, Houston, TX 77030 USA

**Keywords:** Tumor microenvironment, iScores, Integrated scores, Cell type estimation, Deconvolution, Pan-cancer analysis, Immune cells, Stroma, Survival, Tumor progression, Somatic mutations, Computational biology and bioinformatics, Cancer, Cancer microenvironment

## Abstract

Traditional gene expression deconvolution methods assess a limited number of cell types, therefore do not capture the full complexity of the tumor microenvironment (TME). Here, we integrate nine deconvolution tools to assess 79 TME cell types in 10,592 tumors across 33 different cancer types, creating the most comprehensive analysis of the TME. In total, we found 41 patterns of immune infiltration and stroma profiles, identifying heterogeneous yet unique TME portraits for each cancer and several new findings. Our findings indicate that leukocytes play a major role in distinguishing various tumor types, and that a shared immune-rich TME cluster predicts better survival in bladder cancer for luminal and basal squamous subtypes, as well as in melanoma for RAS-hotspot subtypes. Our detailed deconvolution and mutational correlation analyses uncover 35 therapeutic target and candidate response biomarkers hypotheses (including *CASP8* and *RAS* pathway genes).

## Introduction

The tumor microenvironment (TME) is a dynamic ecosystem consisting of various cell types and processes that play a crucial role in tumor initiation, growth, progression, metastasis, and response to therapy^1–4^. A detailed characterization of the TME and its association with genomic and clinical features has yet to be described. Such an analysis can deepen our understanding of tumor biology and resistance mechanisms (e.g., immune escape and suppression), may guide biomarker discovery and help identify therapeutic strategies for cancer.

The gold-standard to study TME is single cell transcriptomics and spatial proteomics or transcriptomics^5,6^. These approaches currently have limitations ranging from low-resolution (spatial transcriptomics), limited number of markers (spatial proteomics) to potential loss of cell types during sample preparation (single cell RNAseq (scRNA-seq)), and high cost^7^. As an alternative, computational methods can be used to deconvolve a tumor sample from its bulk gene expression profile (which is a mixture of tumor and TME cells)^8,9^. The advantage of such methods is that they can be applied to thousands of existing gene expression profiles from tumor tissues and can provide a comprehensive assessment of the TME. Numerous TME studies have applied computational deconvolution to large expression datasets to assess immune and, to a lesser extent, stromal components of the TME^10–17^. These studies have identified broad patterns of immune infiltration across multiple cancer cohorts, and in some cases associated these patterns with prognostic significance^10–17^. These studies typically use one computational deconvolution technique, which limits the number of cell types estimated and may suffer from systematic biases. As a result, multiple constituents of the TME, how they correlate with each other, form patterns of infiltration and predict outcomes remain poorly understood. Additionally, there has been little study of how cancer genomics sculpt TME patterns, which requires analysis of thousands of tumors.

**Fig. 1:**
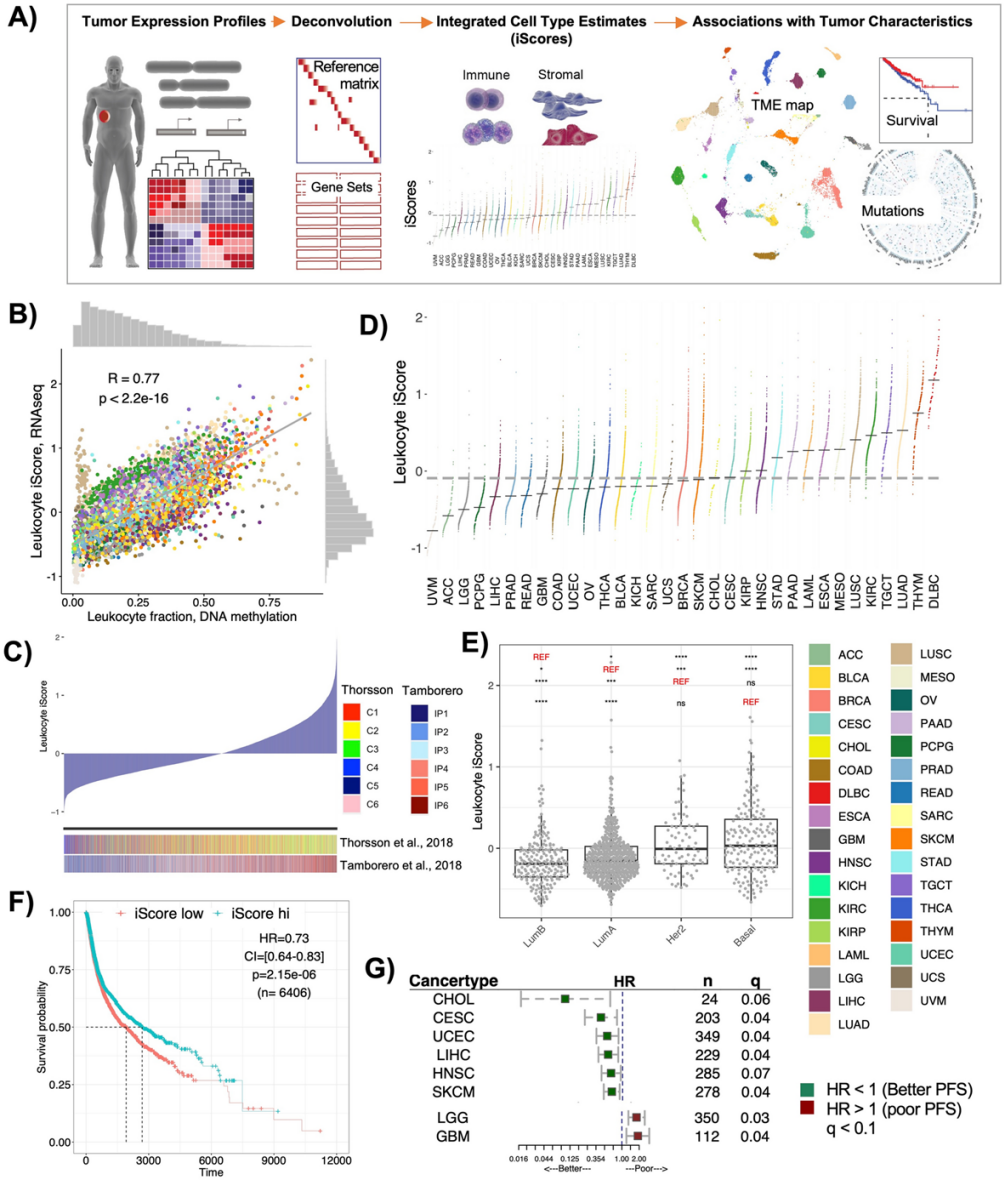
Deconvolution of tumor microenvironment (TME). (**A)** Overview of the study workflow. Tumor expression profiles were deconvolved using nine tools (either reference matrix based, or gene set based) into various cell types of the TME. The individual scores from each tool were normalized and combined as iScores. The association of these iScores were studied with cancer type, patient survival and driver mutation profiles. (**B)** Pan-cancer correlation between leukocyte iScores from GEPs and leukocyte fractions from DNA methylation arrays. Samples are colored by cancer type. (**C)** Ordered leukocyte iScores compared to the immune subtypes identified in two previously published studies. C1-6 are immune subtype clusters from Thorsson et al., (C1: Wound healing, C2: IFNg dominant, C3: Inflammatory, C4: Lymphocyte depleted, C5: Immunologically quiet, C6: TGFb dominant) and IP1-6 are immune phenotype clusters from Tamborero et al., where 1 is least cytotoxic and 6 is most cytotoxic immunophenotype. (**D**) Distribution of leukocyte iScores in 33 cancers ordered by median iScores. Dark gray dashes indicate cancer specific medians, gray dotted line indicates pan-cancer median. (**E)** Distribution of leukocyte iScores across four BRCA subtypes. Significant p-values (< 0.05) are shown with asterisk (Mann–Whitney test). ns is non-significant, and REF is reference subtype used for comparison with other subtypes. (**F**) Pan-cancer Kaplan–Meier survival curves for PFS in patients stratified by leukocyte iScores. High is upper tertile and low is bottom tertile of the leukocyte iScores. Low iScores are reference group. HR is hazard ratio and p is log-rank p-value from multivariate Cox-ph regression models adjusted for cancer type, tumor localization, age at the time of diagnosis and gender. (**G**) Cancer specific forest plots for PFS in patients stratified by individual leukocyte iScores. Low iScores are reference group Threshold of significance for FDR corrected p-values from multivariate Cox-ph (q) is 0.1.

To address the limits of each individual deconvolution tool, we developed a strategy to combine results from nine different supervised deconvolution tools into one integrated score (iScore) per cell type. The concept of combining different deconvolution sources has been recently attempted^18,19^. One such approach focused on merging gene signatures from different tools, which were later used to deconvolve 15 immune cell-types using a single method called ssGSEA^18^. Instead, we aimed to integrate cell type compositions inferred from each tool. Our computationally inferred TME landscape consisted of 79 different cell types/biological processes, including immune and stroma cell types. Applied across 33 distinct TCGA cancer types, we identified numerous associations between TME and patient survival and genomic features. Globally, our deconstructed TME profiles segregated tumors into 41 distinct clusters, predominantly driven by cancer type and patterns of leukocyte infiltration (Fig. 1A). Unlike other reports, these results using more cell types emphasize how each cancer type, when viewed comprehensively, has a unique TME architecture.

## Results

### Integrated scores combine cell type estimates from different tools

We applied nine deconvolution tools to gene expression profiles (GEPs) of 10,592 tumor and normal tissues (Fig. 1A, Tables S1, S2). The deconvolved cell type estimates were standardized across all samples. Standardized estimates were averaged across all tools resulting in one integrated score (iScores) per cell type. This approach led to quantification of 79 cell types (immune, stromal, progenitor and stem cells) (Table S3). Since TME conditions such as hypoxia and angiogenesis may influence tumor growth and metastasis, we also quantified 24 processes related to TME function and sensitivity to immunotherapy (Table S3). We calculated two aggregated scores: one average score for all leukocytes (leukocyte iScores) and another for all stroma cell types (Stromal iScore).

We validated our approach against measures of total immune content from different sources. First, we compared the leukocyte iScores with leukocyte fractions obtained from DNA methylation profiles and found a significantly positive pan-cancer correlation between them (r = 0.77) (Figs. 1B, S1A). 28 of the 30 cancer types had a median correlation of 0.80 ± 0.13, with the exceptions of LUSC and UCS (Fig. S1B). This variation in correlations across cancer types can, in part, be explained by tissue-specific methylation probes, as DNA methylation patterns can vary by tissue type. When compared to tumor purities, leukocyte iScores had strong negative correlations with tumor purities inferred from both RNA-seq (ESTIMATE^20^ r = − 0.83) and DNA-seq (ABSOLUTE^21^ r = − 0.60) (Table S4). Percentage of tumor infiltrating lymphocytes (TILs) quantified from H&E-stained slide images, and abundance of T-cell receptors quantified from bulk RNA-seq also had positive correlations with corresponding aggregated lymphocyte and T-cell iScores (r = 0.34 and 0.65, respectively) (Table S4). We found that iScores had more highest correlations than individual estimates from each tool (Fig. S1A, Table S4).

To compare iScores with previously published TME deconvolution, we selected two studies, each of which had clustered the TCGA tumors into six distinct immune subtypes^14,17^. The median leukocyte iScores increased from least cytotoxic to most cytotoxic immunephenotypes^14^ and were highest in interferon gamma (IFNg) and inflammatory clusters (C2,C3) and lowest in immune quiet cluster (C5)^17^ (Figs. 1C, S1C), showing consistent patterns with published immune subtypes of cancer (Anova p < 2e−16).

In addition to leukocyte iScores, we tested iScore accuracy for individual cell types. We created three sets of 1,000 pseudobulks each by mixing known proportions of labelled cell types from scRNA-seq (kidney n = 10, endometrial n = 6 and lung n = 5) (Fig. S1D). All pseudobulks were deconvolved using the iScore approach. The cell type iScores showed positive correlations to original mixing fractions, with highest immune cell correlations found for macrophages, mast cells, monocytes, Natural Killer (NK) cells, plasma cells, CD8 + T cells and regulatory T cells (Tregs) (r ≥ 0.5) (Fig. S1E). Compared to individual tools, iScores had the highest average correlations with original mixing fractions for all cell types deconvolved from pseudobulks (Fig. S1F). We also used pseudobulk data to compare iScores with two published deconvolution aggregation methods: ConsensusTM and Decosus^18,19^. In 70% of all comparisons, iScore correlations with ground truth were better than or identical to those achieved by either ConsensusTME or Decosus. In contrast, ConsensusTME showed better or equal correlations in only 26% of comparisons, and Decosus in 54% (Table S5). Altogether, these computational comparisons suggest that our integrative approach to cell type deconvolution can provide improved results across numerous cell types.

### Leukocyte abundance is heterogenous and correlates with tumor progression

Leukocyte iScores distributions varied extensively across and within the 33 cancers; highest in hematologic cancers (e.g., DLBCL, THYM) and lowest in cancers at immune privileged sites^22^ (e.g., UVM, LGG) (Fig. 1D). We analyzed the leukocyte abundance by tumor localization in skin cutaneous melanoma (SKCM), the only TCGA cohort with more metastatic tumors (metastatic n = 367, primary n = 103). In SKCM, metastatic tumors localized to lymph nodes had higher leukocyte abundance compared to tumors at primary or other metastatic sites (Fig. S2A). Metastatic lymph node sites are known to develop an immunosuppressive TME to allow for tumor growth, and higher leukocyte iScores for these sites suggests a possibility of clinical benefit from immune checkpoint immunotherapies that circumvent immune evasion^23,24^. Cancer-dependent differences in leukocyte abundance also existed between tumor and adjacent normal tissues *e.g.,* leukocytes were significantly higher in tumors relative to adjacent normal in renal cancer KIRC (p < 2.2e^−16^) (Fig. S2B).

Leukocyte abundance varied among cancer subtypes. Leukocytes were higher in seminomas compared to non-seminoma testicular cancer (TGCT)^25^; higher in stroma-rich and basal squamous bladder cancer compared to the luminal papillary subtype (LumP) in BLCA^26^ (Fig. S2C). Breast cancer (BRCA) luminal subtypes (LumA, LumB) had significantly lower levels of leukocytes compared to Basal-like and HER2-Enriched subtypes (Fig. 1E), and within HER2-Enriched subtypes, leukocytes were higher in triple negative breast cancer (TNBC) (Fig. S2D). These findings indicate substantial diversity in TME composition within the same cancer type and subtype.

We tested the prognostic relevance of leukocyte levels in the TME. Tumors with high leukocyte iScores showed lower risk of progression pan-cancer (hazard ratio HR_adj_ = 0.73, p = 2.15e−06, n = 6406) (Fig. 1F). Except for brain cancers (GBM, LGG), cancer cohorts that showed survival associations with leukocyte abundance showed positive correlations, i.e., more leukocytes were associated with better outcome (Fig. 1G). One explanation for the brain cancer finding is that leukocyte infiltrates in the brain can promote the release of immunosuppressive cytokines that may disrupt the anti-tumor immune activity^27^.

### Heterogeneity in immune cell composition and risk of progression

**Fig. 2:**
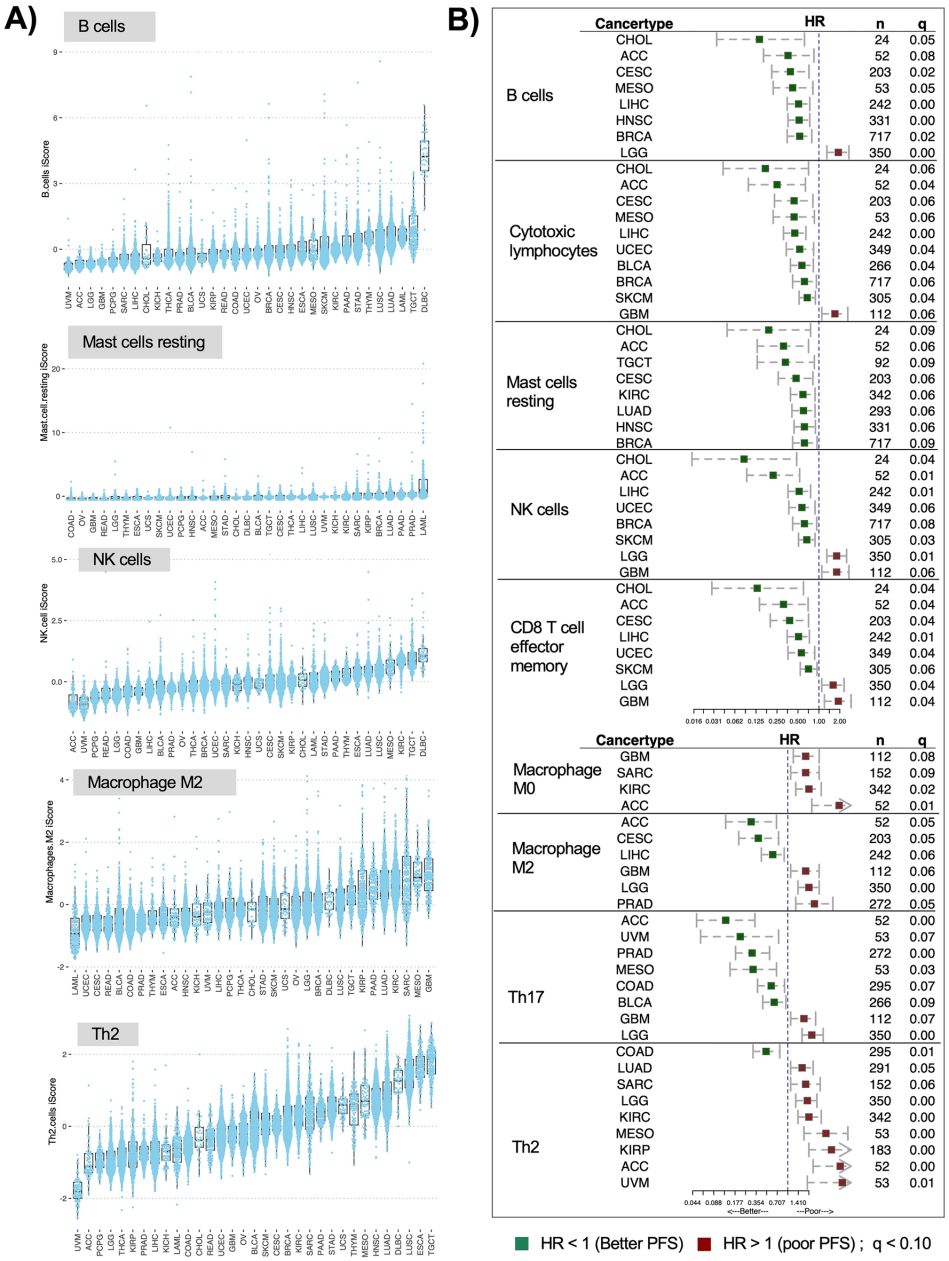
Immune cell type specific iScores and their association with PFS. (**A)** Distribution of cell type iScores ordered by cancer specific medians. (**B)** Forest plots for PFS in patients stratified by individual cell type iScores (reference group: low). HR is hazard ratio and q is FDR corrected (for each cell type) log-rank p-value from multivariate Cox-ph regression models for each cell type across. Visualization is restricted to cancer types significant at q < 0.1.

Like leukocytes, individual immune cells were usually most abundant in hematologic cancers, with some exceptions *e.g.,* highest T helper 2 (Th2) in TGCT, Th17 in KICH, and dendritic cells in LUAD (Figs. 2A, S3A, Table S6). The distribution patterns differed within macrophage subclasses: uncommitted M0 and pro-inflammatory M1 macrophages were highest in DLBC but polarized anti-inflammatory M2 macrophages were highest in GBM (Fig. 2A, Table S6). These differences are likely due to different signaling molecules in the TME that determine macrophage polarization, for example the CSF-1R signaling in gliomas promotes polarization of macrophages towards M2 subclass^28^.

Survival analysis highlighted cancer dependent relationships between progression free survival (PFS) and immune cell abundance (Figs. 2B, S3B). Overall, 51 immune cell types had significant positive or negative associations with risk of survival in 22 cancer types (Fig. S3B, Table S7). Cytotoxic T lymphocytes, B cells, resting mast cells, NK cells, CD8 T effector memory cells, and Th17 cells had a predominantly positive association with PFS (Fig. 2B). Low grade gliomas (LGG) had positive PFS associations with more plasma cells (HR = 0.35, CI = 0.25–0.50, q = 4e−07) and resting dendritic cells (HR = 0.59, CI = 0.43–0.83, q = 0.03) (Fig. S3B), but high-grade glioblastomas (GBM) only had negative PFS associations with immune abundance. Macrophages (HR = 2.0, q = 0.02), including M0 and M2 macrophages (HR = 1.8, q < 0.1) were among the cell types most associated with increased risk of progression in GBM.

Tregs are commonly regarded as immune suppressors^29^. In our analysis, more Tregs associated with significantly worse prognosis only in renal clear cell carcinoma, (KIRC; HR = 2.5, CI = 1.7–3.8, q = 5e−04) (Fig. S3B, Table S7). Thus, KIRC patients may particularly benefit from inhibiting Tregs, *e.g.,* by blocking immune checkpoint *VISTA* thereby reducing levels of Tregs^30^. We also found that Th2 cells had negative prognostic associations with most cancers (n = 8), except for in COAD (Fig. 2B). Previous studies suggest that Th2 can exhibit both anti-tumor activity (via cytokine IL-5 mediated recruitment of eosinophils), and pro-tumor activity (via cytokine e.g., IL-4 mediated suppression of pro-tumor Th1 cells)^31,32^.

Since BRCA is the largest cohort in the TCGA, we were able to conduct a subtype analysis of CD8 + cytotoxic T lymphocytes (CTLs) across different subtypes of this cancer. The HER2-Enriched and basal-like subtypes had significantly higher CTLs compared to LumA and LumB (Fig. S4). We identified a significantly lower risk of progression for BRCA tumors with more CTLs, especially for the HER2-Enriched subtype (reference: LumA; HR = 0.13, p = 0.02; n_Her2_ = 54, n_LumA_ = 454) (Table 1).

**Table 1:** PFS for BRCA subtypes and cytotoxic lymphocytes iScores.

Characteristic	HR^1,2^	SE^2^	95% CI^2^	p-value
Cytotoxic Lymphocytes * BRCA Subtype^3^				
Cytotoxic Lymphocytes * Basal	0.65	0.48	0.25, 1.68	0.40
Cytotoxic Lymphocytes * Her2	0.13*	0.90	0.02, 0.73	0.02
Cytotoxic Lymphocytes * LumB	1.21	0.48	0.41, 3.10	0.70

^1^ *p < 0.05; **p < 0.01; ***p < 0.001.

^2^ HR = Hazard Ratio, SE = Standard Error, CI = Confidence Interval.

^3^ Reference subtype LumA.

CD8 + T cells are central to immune checkpoint immunotherapies^33^, so we tested combinations of other cell types with CD8 + T cells. Low CD8 + T cells combined with high macrophages, high Th2 cells, or high hypoxia levels associated with higher risks of progression (HR > 1, p < 0.05) (Table S8). Conversely, high CD8 + T cells combined with low Th2 associated with lower risks of progression (Table S8). In addition to revealing new hypotheses, these results suggest that risk models capturing cell type combinations can be valuable in predicting for patient outcomes.

### Stromal composition and immune-stroma interactions in context of progression risk

**Fig. 3:**
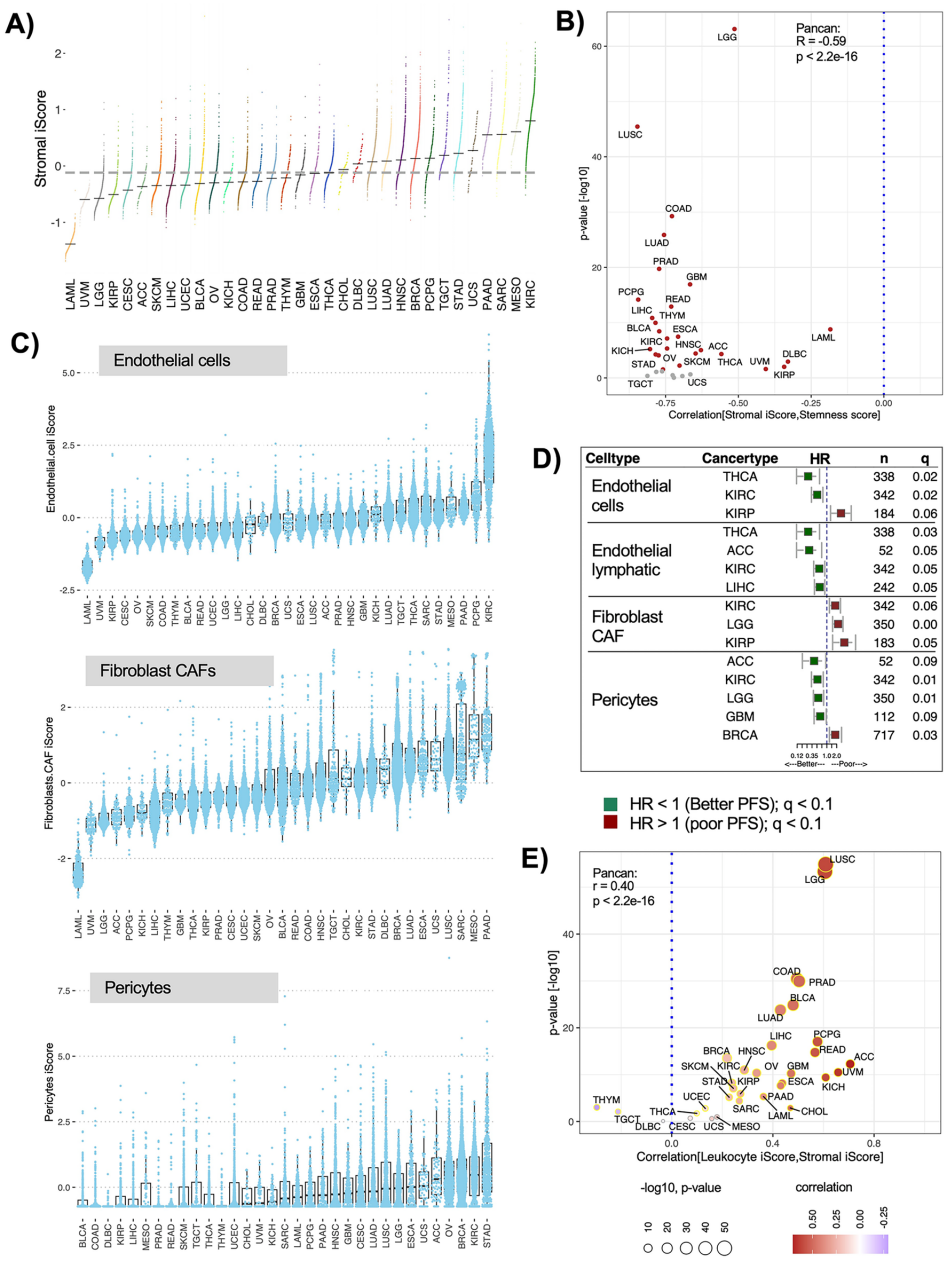
Stroma specific iScores and their association with PFS. (**A)** Distribution of stromal iScores across 33 cancer types ordered by their cancer specific medians. The gray dashes indicate cancer specific medians, gray dotted line indicates pan-cancer median. (**B)** Pan-cancer correlations between stromal iScores and cancer stemness scores for each cancer type plotted against the p values (y-axis) for significance of correlation estimate. Significant p-values are colored red. (**C)** Distribution of stromal cell type iScores ordered by cancer specific medians. (**D)** Forest plots for PFS in patients stratified by individual stromal cell type iScores (reference group: low). HR is hazard ratio and q is FDR corrected (for each cell type) log-rank p-value from multivariate Cox-ph regression models for each cell type across. Visualization is restricted to cancer types significant at q < 0.1. See also Fig. S5. (**E)** Pan-cancer correlations between leukocyte and stromal iScores for each cancer type. x-axis is correlation estimates and y-axis is p values from correlation test. Significant p-values are circled in yellow.

Compared to immune infiltration, stromal composition of the TME (including fibroblasts, endothelial cells, pericytes) is less understood. In our analysis, highest stroma content was identified in renal clear cell carcinoma (KIRC), with a comparatively much lower content in the related renal papillary carcinoma (KIRP) (Fig. 3A). One explanation for this paradoxical difference between the two renal cancer types is that KIRP has a rich papillary architecture while KIRC is comparatively more vascular as shown by the higher expression of vascular marker genes (*CD34* and *CD31*) in KIRC compared to KIRP (p-values < 2.2 e−16) (Fig. S5A). Pancreatic cancer (PAAD) had predictably high stroma^34^ (Fig. 3A). Stroma content also varied by tumor localization and subtype (Fig. S5B-C). Stroma associated negatively with cancer stemness^35^ for all cancer types, suggesting that high stroma is perhaps indicative of high mesenchymal differentiation (pan-cancer r = − 0.59) (Fig. 3B).

Globally, stromal iScores did not associate with survival (HR = 1.1) (Fig. S5D) but individual stromal cell types showed significant associations with PFS. For example, endothelial cells, highest in KIRC (Fig. 3C), associated with significantly lower risk of progression (Fig. 3D). Pericytes associated with lower risk of progression in ACC, KIRC, and the brain cancers (LGG, GBM) but higher risk of progression in BRCA (Fig. 3D). Cancer associated fibroblasts (CAFs) had negative associations with PFS for KIRC, KIRP and LGG (Fig. 3D).

We evaluated stroma in the context of immune cell types. The stroma iScores correlated positively with leukocyte content (r = 0.40) except for in THYM and TGCT (Fig. 3E). We then tested survival associations for concomitant variations in CD8 + T cells and stroma cell types. Low CD8 + T cells combined with low endothelial cells associated with higher risks of progression (HR > 1, p < 0.05) while high CD8 + T cells combined with low CAFs associated with lower risks of progression (Table S8). These results suggest a role of immune-stroma interactions in regulating tumor growth in the TME.

### Global TME map identifies subtype dependent differences in bladder and skin cancers

**Fig. 4:**
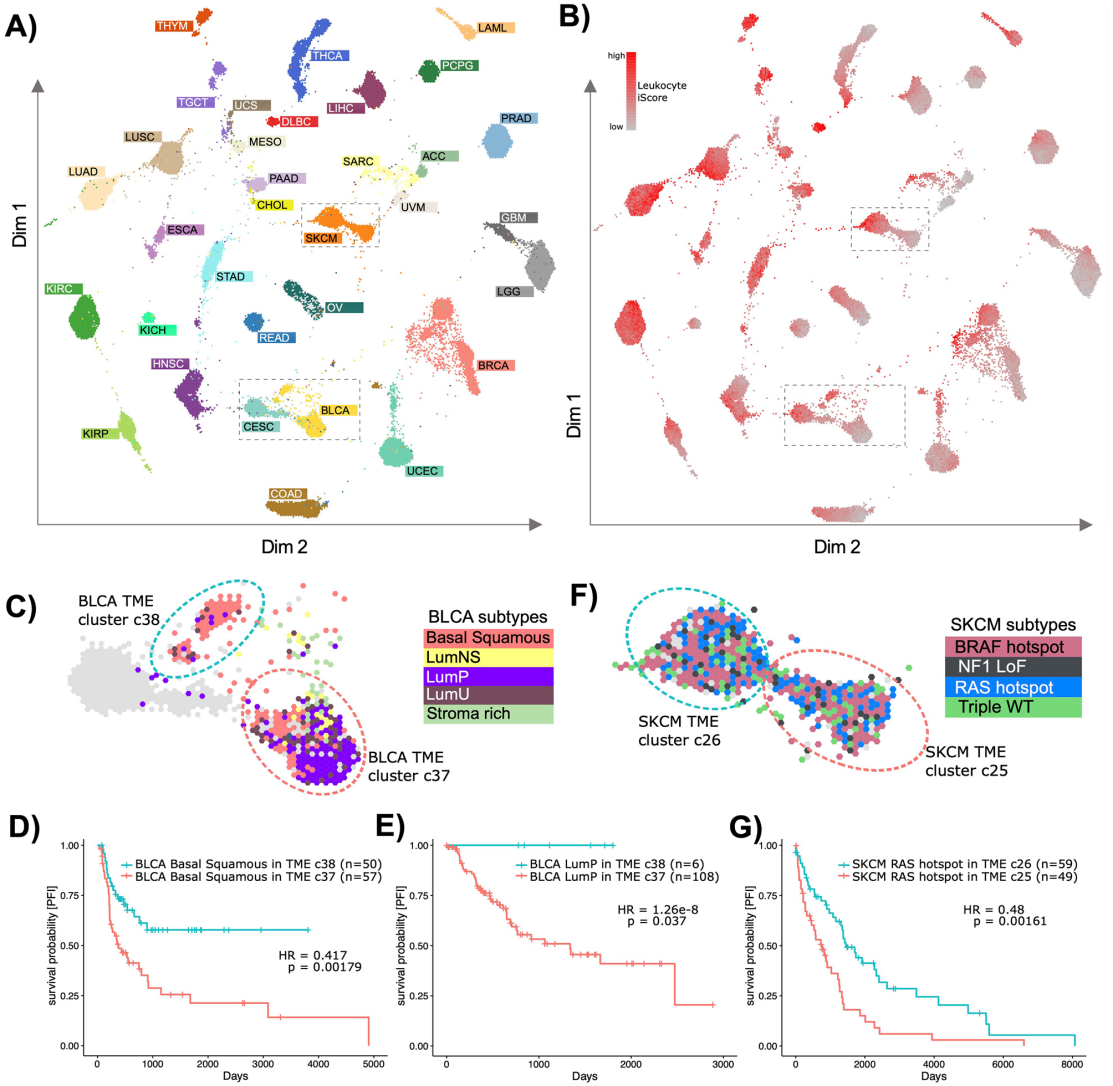
TME Map using cell type iScores. (**A)** Two-dimensional Tumor Map projection of samples using sample similarities in cell type iScores, clustered using HDBSCAN clustering method with a minimal cluster size of 20. Each sample is colored by its cancer type. (**B)** TME map colored by leukocyte iScores. (**C)** BLCA focused part of TME Map as indicated by a dashed box in panels A and B. Samples are colored by BLCA subtype. LumNS = luminal non specified, LumP = luminal papillary, LumU = luminal unstable. clusters c37 and c38 are circled in red and blue, respectively. Kaplan–Meier survival curves for PFS between clusters c37 and c38 for BLCA. (**D)** Basal Squamous subtype and (**E)** luminal papillary subtype. HR is hazard ratio and p is p-value from the Cox-ph regression models. (**F)** SKCM focused part of TME map as indicated by a dashed box in panels A and B. Samples are colored by SKCM mutational subtype (LoF = Loss of function; WT = wild type). clusters c25 and c26 are circled in red and blue, respectively. (**G)** Kaplan–Meier survival curves for PFS between clusters c25 and c26 for SKCM RAS hotspot subtype, corrected for tumor localization.

We sought to leverage iScores to identify global patterns of microenvironment similarities among cancer types. We took a comprehensive approach to create a pan-cancer TME map from unsupervised clustering of all 79-cell type iScores. We found that the tumors segregated into 41 distinct clusters (Fig. S6A) in the TME map, primarily based on cancer type and patterns of overall immune infiltration (Fig. 4A, B).

To identify clinically relevant clusters for each cancer, we applied survival analysis to differently clustered tumors from the same cancer. We reviewed the ones with most significant associations with risks of progression. We identified two bladder cancer (BLCA) clusters—an immune rich c38 and immune depleted c37 (leukocyte iScore p-value = 10e−34) (Fig. 4C, Table S9). c38 was characterized by higher inflammation and adaptive immune response: IFNg, T-cells, Checkpoint, CD45 positive cells, and Major Histocompatibility Complex iScores compared to c37 (Table S9). The median mutation load was also significantly higher in c38 compared to c37 (p-value < 0.01) (Fig. S6B-C). The immune-rich c38 cluster mainly consisted of basal squamous subtype tumors (n = 50, 79%), with a small fraction of luminal subtype tumors (luminal-papillary LumP, n = 6, luminal unstable LumU, n = 7). Conversely, the immune depleted cluster c37 was predominantly LumP (n = 108, 45%), along with basal squamous (n = 57) and other subtypes (n = 68) (Fig. 4C).

Basal squamous tumors in c38 (immune-rich) associated with significantly lower risk of progression compared to those in c37 (immune-depleted) (HR = 0.42, p = 0.002) (Fig. 4D, Table S9). The same was noted for LumP tumors (Fig. 4E), but with limited interpretability because of the biased sample sizes (n = 6 *vs*. 108). These analyses show that even within the same BLCA subtype, we were able to identify a subset of tumors with high T cell recruitment and high mutation load (likely indicative of high neoantigen load ^36^), which also associated with lower risk of progression.

We found two major clusters in SKCM, immune-rich c26 and immune-depleted c25 (c26 n = 217 (198/217 metastatic), c25 n = 183 (127/183 metastatic), leukocyte iScore p-value = 10e-72) (Figs. 4F, S6D). We found that only the *RAS*-hotspot subtype tumors in c26 (immune-rich) associated with more than two-fold lower risk of progression compared to those in c25 (immune-depleted) (HR = 0.47, p = 0.001) (Fig. 4G, Table S9). There were no differences in median mutation load between the two clusters (Fig. S6E) but the *RAS*-hotspot tumors in c26 had significantly higher immune checkpoint gene iScores compared to c25 (p = 1.97e−29). Given the success of immune checkpoint inhibitors in *KRAS* mutated lung tumors with high *PD-L1* expression^37^, it can be hypothesized that the immune rich *RAS*-hotspot melanomas (also characterized by high immune checkpoint iScores) may benefit from similar immunotherapies.

### Somatic alterations in driver genes associate with immune-stromal contexture

The relationship between the TME and somatic mutations remains poorly defined. This is an important gap to address; a better understanding of such relationships could reveal opportunities to target tumor cell proteins to modulate TME for therapeutic advantage. Therefore, we studied relationships between 299 driver mutations^15^ and patterns of TME composition using linear regression models.

**Fig. 5:**
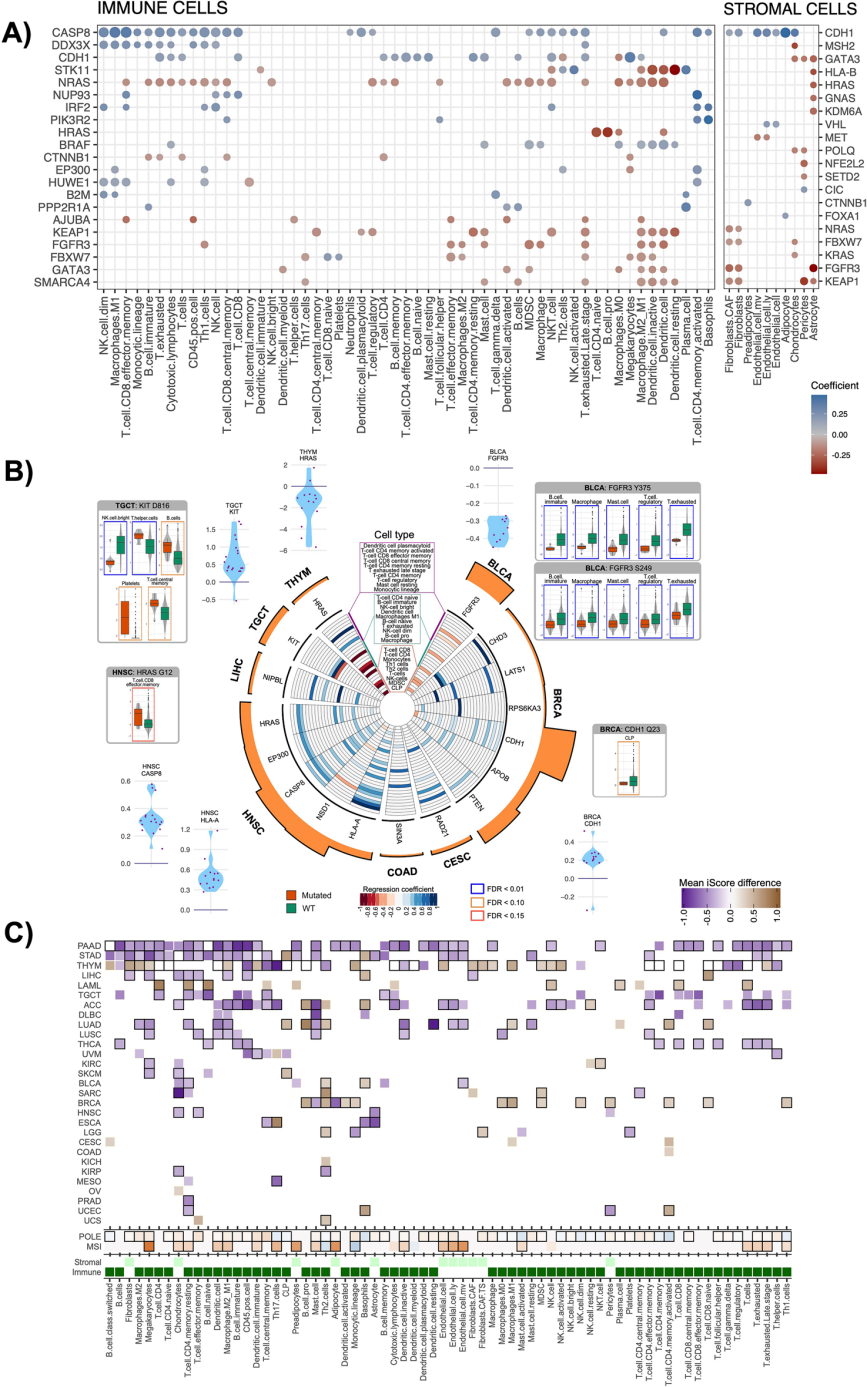
Associations of somatic alterations with cell types. (**A)** Pan-cancer regression coefficients (|coef|> 0.1) for selected driver genes that are associated with either 15 immune or 3 stromal cell types for more than 5 tumors per cancer cohort. Thresholds: |coef|> 0.1, FDR < 0.1. (**B)** Circos plot for cancer-specific regression coef in immune cell types for driver genes mutated in more than 5 tumors per cancer type (|coef|> 0.1, FDR < 0.1). Histogram around the circos plot indicates the number of tumors mutated for the corresponding gene. Blue violin plots show genes mutated in > 5 tumors and associated with > 8 immune cell types per cancer. Boxplots show specific variants of significantly mutated genes within a cancer type (n > 5 tumors/cancer type). (**C)** Cancer-specific analyses to show differences in means of iScores for tumors segregated by high or low mutation loads bi-clustered using QUBIC (upper panel, black outline indicates FDR < 0.05), Cancer-specific regression coefficients (coef) for associations between MSI or POLE mutation status and cell type iScores (middle panel), and Leukocyte or stromal cell status (lower panel).

We identified 35 driver genes that significantly associated with >  = 3 distinct cell types (q < 0.1) (Table S10). Amongst the top associations were genes involved in the IFNg response (*NUP93, CASP8, IRF2, B2M*), TGF-beta signaling (*CDH1, CTNNB1*), *PDGFR*-beta signaling pathway (*NRAS, PPP2R1A, PIK3R2, BRAF, HRAS*), and *RAS* signaling pathway (*NRAS, HRAS*) (Fig. 5A). While mutations in genes from the immunosuppressive *TRAIL* signaling pathways (*CASP8, PIK3R2*) associated positively with higher levels for most cell types (q < 0.05) (Fig. 5A), *NRAS, KEAP1*, and *FGFR3* mutations associated negatively with a wide variety of cell types (q < 0.05) (Fig. 5A, Table S10). Roles of *CASP8* and *NRAS* in immune infiltration have been reported earlier^17,38^. Here we show that *CASP8* mutations positively associate with as many as 26 different immune cell types (Fig. 5A), suggesting a broader mechanism adopted by tumors to escape immune surveillance. In contrast, *NRAS* mutations that negatively associated with 25 distinct immune cell types may suggest its role in immune exclusion.

We tested the cell type-mutation relationships for individual cancers (Figs. 5B, S7A, Table S11–S12). We identified prominent negative associations of *FGFR3* mutations (especially p.S249, p.Y375) with 11 immune and three stromal cell types in bladder cancer, suggesting FGFR3 may have a role in establishing an immunosuppressive TME in bladder cancer (Figs. 5B, S7A, Table S12). In contrast, in TGCT, the *KIT* p.D816 variant associated positively with higher T helper cells, but negatively with NK bright cells (FDR < 0.01) (Fig. 5B).

Tumor mutation burden (TMB) and T-cell levels are considered important for predicting responses to immunotherapy^39^, hence we tested how these features correlated with each other. Previously, TMB was known to show little correlation with T-cells in melanomas and HNSC^40^. In our analyses, we found no pan-cancer correlations between TMB and leukocyte or stromal iScores (r = 0.12 and − 0.08 respectively), and correlations varied by cancer type (Fig. S7B-C). In a pan-cancer analysis focused only on high (top 25%) and low (top 25%) TMB tumors, high TMB group had higher median leukocyte iScores compared to low TMB group (Fig. S7D). Cancer specific patterns also emerged. For example, PAAD had low mean iScores in high TMB group for all significant comparisons (n = 39) (Fig. 5C). In thymoma, most cell types of lymphocytic lineage had negative associations with TMB, while cell types of monocytic lineage had positive associations (Fig. 5C).

## Discussion

We developed a systematic approach to leverage existing deconvolution tools and present a comprehensive TME map using diverse algorithms, cell types and gene markers to achieve a consensus cell type estimate that was validated against direct and indirect measures immune content from other sources (leukocyte fractions from DNA methylation arrays and tumor purity estimates from high-throughput datasets). Our iScores showed strong concordance with scRNA-seq derived pseudobulks. While our results support previous findings showing our ability to accurately infer TME composition and biological relevance, we noted many new observations. This resource may help understand heterogeneity in responses to checkpoint-based immunotherapies; the higher immune infiltrates in tumors compared to its adjacent normal tissue in renal clear cell cancers (KIRC) may partially explain this cancer’s susceptibility to immunotherapy. We found inconsistent correlations between mutation loads and immune infiltration, suggesting that TMB is not always a driver of immune infiltration and may not be a perfect independent predictor for immunotherapy outcomes^40–42^. At individual mutation levels, we identified pan-cancer and cancer specific associations between specific driver mutations and cell type iScores, that may partially explain resistance mechanisms activated by tumors such as escape from immune surveillance (*e.g., CASP8*) or immune exclusion (*e.g., NRAS*). In summary, our results systematically characterize the immune and stroma landscape of the TME in the TCGA tumors, and associations with risk of progression.

## Methods

### Datasets

#### Datasets for deconvolution

The datasets used for deconvolution were downloaded from the Genomic Data Commons (GDC) Data Portal (https://portal.gdc.cancer.gov); these comprised of RNA-seq based gene expression profiles normalized as Fragments Per Kilobase of transcript per Million mapped reads (FPKMs). These datasets had been harmonized under the GDC guidelines: realigned to the GRCh38 genome build and reprocessed using their standardized pipelines (https://gdc.cancer.gov/about-data/gdc-data-harmonization). Out of the 11,093 samples downloaded in total from the GDC, we selected a final list of samples based on the merged quality annotation file put together by the Pan Cancer Atlas Consortium (http://api.gdc.cancer.gov/data/1a7d7be8-675d-4e60-a105-19d4121bdebf). Briefly, we removed samples which either failed an expert pathology quality control review or whose aliquot barcodes were labeled as ‘do not use’. We also excluded samples for which a pathology report was unavailable. After these filtering steps, we retained a total of 10,592 samples that belong to 33 cancer types and included 9,892 tumor and 700 tumor adjacent normal tissues.

#### Datasets for validation

The primary dataset used for validation of leukocyte estimates was leukocyte fractions quantified from the DNA methylation arrays^17^. The comparison was performed for 9,495 samples that had both RNAseq and DNA methylation-based leukocyte quantifications, and these samples were used for further validations. The tumor infiltrating lymphocytes (TIL) quantification from TCGA H&E slides imaging data, inferred using Convolutional Neural Networks, was obtained from a recent publication^43^. DNA based purities were obtained from ABSOLUTE^21^ and RNA-seq based transcript proportions were obtained from DeMixT^16,44^**.** The tumor purity was also inferred from RNA-seq FPKMs using ESTIMATE^20^. The analysis was done using ‘estimate’ package (v1.0.13) in R. The package was run with the platform type input parameter set to “Illumina” and all other parameters set to default.

#### T cell receptor beta variable (TRBV) region quantification

The TRBV regions were quantified using a previously described method^45^. Briefly, the RNA sequencing reads were submitted for a nucleotide BLAST-mapping against a custom database comprised of the TRBV genes downloaded from the IMGT (http://www.imgt.org). These reads that met the BLAST-expected value cutoffs for each β variable gene were counted and normalized to counts per million sequenced reads. This data set was also used for comparison with the leukocyte iScores and estimates from individual tools.

#### TCGA data on clinical information, molecular subtype, and somatic alterations

The clinical data including patient survival information was obtained from Liu et al., which provided progression free survival (PFS) for 32 cancer types (except for LAML)^46^. The molecular subtype assignments for the TCGA cohort were obtained from the Tumor Molecular Pathology (TMP) Analysis Working Group (Genomic Data Commons, *manuscript submitted*). Somatic mutations used in study were obtained from the previously reported mutation calling harmonization effort, performed by the Pan Cancer MC3 Consortium^47^. The 299 driver genes were obtained from a recent Genomic Data Analysis Network (GDAN) publication regarding the identification of cancer driver genes^15^*.* Cancer stemness scores were obtained from another pan cancer effort by the TCGA consortium^35^. A curated resource for matched tumor and adjacent normal tissue was carefully compiled by manual inspection (Alexander Lazar, UT-MD Anderson Cancer Center, Texas, USA). Information regarding the estrogen (ER), progesterone (PR), and Her2 receptor status (positive or negative) for breast tumors was obtained as follows: ER and PR quantifications were strictly generated through Immunohistochemistry (IHC), while for the HER2 receptor status determination the preferred method for quantification was IHC, but fluorescence in situ hybridization (FISH) and copy numbers were used for cases where IHC was noninformative to determine the Her2 status.

### Deconvolution methods

We selected nine different commonly used methods of deconvolution based on following three criteria: (1) ability to deconvolve using bulk RNA-seq gene expression data as input, (2) ability to report total cell type content (measures of abundance) in a tumor’s microenvironment, and (3) tools that only provide relative fractions of cell types were not considered. The latter was crucial to allow for integration of outputs from different methods because relative proportion from each method is a function of the number of cell types deconvolved by that method. Based on these criteria, we selected nine methods of deconvolution in total: eight published tools Cibersort^48^, Cibersortx ^49^, MCP Counter^50^, xCell^51^, EPIC^52^, TIMER ^51^, QuantiSeq^53^ and ssGSEA^54,55^, and one based on a new application of sparse group lasso^56^ (SGL) model to deconvolve the tumor microenvironment (https://github.com/drisso/deconsgl). All tools provided deconvolution estimates for 33 cancer types except for TIMER which doesn’t provide estimates for LAML.

#### Published tools

All tools were implemented using their respective R packages, using the accompanied marker gene signatures, and with the default parameters, as suggested by the authors of these packages^48–51^. Cibersort was run on an ‘*absolute*’ mode with quantile normalization disabled (as recommended by the developers for RNA-seq based inputs). The ‘*absolute*’ mode gives SVR regression coefficients scaled by median expression of genes in the signature divided by median expression of all genes in the input mixture. CibersortX was run using their web interface by first adjusting tumor expression profiles to LM22 signature using B-mode batch correction followed by deconvolution using an SVM model. Single Sample Gene Set Enrichment Analysis (ssGSEA) was implemented using the ‘gsva’ package (v1.30.0) in R using gene lists of immune and stromal cell types which were either previously published^13,57–63^ or publicly available^64–66^. All analysis was performed using R version 3.5.1.

#### Sparse Group Lasso (SGL)

A useful model for the deconvolution of RNA-seq tumor samples has the form of a linear regression model, in which the covariates are the expression signature of the “pure” cell types, and the regression coefficients are the estimated contributions of each cell type to the tumor expression ensemble^48^. One drawback of this model is that the relations between covariates (cell types) are not modeled, possibly leading to collinearity problems when multiple correlated cell types are included. To avoid this problem, we consider a sparse group lasso model^56^, a penalized regression model in which the coefficients are shrunk to zero according to two penalty terms, one that penalizes a group of covariates and one that penalizes each covariate in the group. This model can achieve sparsity both between groups and within each group. More formally, for each sample, our approach consists of minimizing the following quantity:

$$\min_{\beta } \frac{1}{2n}\left\| {y - \sum\limits_{l = 1}^{m} {X^{(l)} \beta^{(l)} } } \right\|_{2}^{2} + (1 - \alpha )\lambda \sum\limits_{l = 1}^{m} {\sqrt {p_{l} } } \left\| {\beta^{(l)} } \right\|_{2} + \alpha \lambda \left\| \beta \right\|_{1} ,$$

where, the $l$ represents the subset of covariates in group $l$, and α is a weight in the convex combination of the regular lasso and the group lasso penalties, X($l$) is the submatrix of *X* (feature matrix)*,* β($l$) is the coefficient vector of group $l$, and $pl$ is the length of β($l$), λ is the tuning parameter. This model was implemented in R using the ‘lsgl’ package (v. 1.3.6) using the LM22 signature^48^. In particular, the covariates (X) are the columns of a validated leukocyte gene signature matrix made of 22 functionally defined human hematopoietic subsets [ref: 10.1007/978-1-4939-7493-1_12] divided into seven manually specified groups. The groups, and the corresponding cell types are B-cells (naïve, memory), T-cells (CD8+, CD4+ naïve, CD4+ memory resting, CD4+ memory activated, follicular helper, T-regs, gamma-delta), NK cells (resting, activated), Macrophages (M0, M1, M2), Dendritic cells (resting, activated), Mast cells (resting, activated), Eosinophilis/Neutrophilis (Eosinophilis, Neutrophilis). The LM22 signature matrix can be downloaded from https://cibersortx.stanford.edu/. Parameter α = 0.5 was used and λ was selected via cross-validation.

### Integrative Scores (iScores) for deconvolved cell types

The individual cell type estimates obtained from different methods were first converted into standardized scores across all samples in the cohort (n = 10,592) as follows: standardized score for each cell type (i) is defined as $i = {\text{ x}}_{{\text{i}}} - \mu \left( {{\text{x}}_{{\text{n}}} } \right)/\sigma \left( {{\text{x}}_{{\text{n}}} } \right)$, where x_i_ is the cell type estimate for the given sample, μ (x_n_) is the mean and σ(x_n_) is the standard deviation of all estimates of the given cell type across all samples (n). To get one score per each cell type, standardized scores corresponding to identical cell types (coming from different methods) were averaged. This averaged score was termed as integrated score (iScore), and one iScore was calculated for each distinct cell type (cell type specific iScores). The iScores for different cell types had a near-Gaussian distribution with mostly a right skew, with means close to zero, the range varied widely across cell types. We also calculated two broader aggregated scores that could capture the overall immune and stromal infiltration in each tumor. These scores were leukocyte iScore and Stromal iScore. Leukocyte iScore is defined as the mean of standardized scores of all deconvolved immune cell types that belonged to either the myeloid or lymphoid lineage. Stromal iScore is defined as the mean of standardized scores of the cell types that were stromal: Fibroblasts, Endothelial cells, Pericytes and Adipocytes. iScores were validated by comparing leukocyte iScores to measures of Leukocyte fractions from DNA methylation arrays and tumor purity using Pearson correlation coefficients. Similarly, the aggregated lymphocyte iScores were calculated and compared to the %TILs and aggregated T-cell iScores were calculated and compared to the TRBV dataset for appropriate comparisons. It is important to note that the standardization strategy used for integration makes iScores ideal for comparisons of individual cell types across samples and cancer types, but not necessarily ideal to study relative percentage of different cell types within individual samples. All correlation coefficients reported in this study use the Pearson method.

### Pseudobulks to validate integrated deconvolution approach

To verify the accuracy of the deconvolution methods, we created pseudobulks from the scRNA-seq profiles obtained from the kidney (n = 10), endometrial (n = 6), and lung (n = 5) tumors. Cell types were labeled using the known gene markers (Table S13) or were predicted using Support Vector Machine (SVM) classification. The validity of selected cell type-specific markers was assessed through cluster visualization (Seurat UMAP) and comparison of marker expression levels in our data to those of the respective immunologic cell types documented in the Blood Atlas^67^. We computationally generated a series of 1000 pseudobulks from the raw expression data, separately and in an identical fashion, for each cohort. Prior to pseudobulk generation, any cell type failing to exhibit expression of its biomarkers consistent with the expected signature was removed from the dataset. For each of the 1000 pseudobulks, 10% of the total available cells were randomly selected. This dataset was then input to ComBat along with corresponding cell types of the LM22 reference dataset for batch correction^48,68^. This step was implemented to coerce the pseudobulk batch expression values into the bulk-like space of the reference batch, which is the expected input for the deconvolution tools. ComBat was run in the non-parametric mode, correcting only the mean of the batch effect with no scale adjustment. To confirm if the marker expression is consistent amongst cell types shared by all three cohorts, we used Seurat^69^ v3. We set dimensionality reduction to dims = 30 on the 5000 most variable features followed by anchor integration and principal component analysis is carried out as described earlier^69^, with all other arguments set to default. Shared cell types are well-correlated amongst cohorts when viewed in a UMAP. Briefly, the 5000 most variable features for each dataset are identified independently, and those features which are repeatedly variable across datasets are employed as anchors for dataset integration. Color-labeling of single cells by cohort depicts satisfactory co-localization amongst most of the clusters (Fig. S1D). Similarly, color-labeling the same cells by cell type (right panel) confirms cells of the same type tend to cluster together. Using this approach, three sets of pseudobulk cohorts were generated, one for each available cancer type. The iScore deconvolution approach was applied to the pseudobulks to deconvolve and obtain estimates of individual cell types in each mixture. The iScores for each cell type were then correlated (using Pearson correlation) with the ground truth for each pseudobulk (i.e., the known proportion of cells in each pseudobulk sample) and the results were reported as correlation heatmaps (restricted to positive correlations) (Fig. S1E). For comparison, deconvolution estimates for pseudobulks were also obtained from previously published methods ConsensusTME^18^ (v 0.0.1.9000) and Decosus^19^ (v 0.1.3), implemented in R as per the tool specifications.

#### Linear Support Vector Machine Classification to label cell types in scRNA-seq

Marker genes were primarily used to label the distinct cell populations in the scRNA-seq samples. However, this strategy resulted in numerous cells without an assigned label. For example, in endometrial tumors, 21% of cells (1,127 out of 5,222 cells) were without an assigned cell type label. To address this gap in labelling, a Linear SVM Classification (SciKitLearn) based approach was applied where the labeled cells were used as a training set for the predictive model. A label balancing strategy was also implemented which limited the training set cell count to the median of cell counts across all cell type—this allowed us to restrict over representation of the more prominent cell types in the model. Multiple models were trained using cross fold validation and the models were then applied to the unlabeled cells to predict their cell type. Ambiguous cell type assignments for each unlabeled cell were identified by calculating the entropy of predicted classifications from the different models.

### Survival analysis

The survival analysis was performed using Cox proportional-hazard regression model (Cox-ph) using the ‘survival’ (version 2.44.1.1) and ‘survminer’ (version 0.4.3) packages in R. The results of survival analysis were visualized as Kaplan–Meier curves using ‘ggsurvplot’ function of the ‘survminer’ package in R. The analysis was conducted to assess differences in progression free survival (PFS) between cases with high versus low cell type iScores; high and low were defined as values being in the top 1/3rd or bottom 1/3rd of the global iScores respectively. All pan cancer Cox-ph models were adjusted for cancer types, sample localization, age, and gender. Tumor stage was also used as a covariate, where available. The p-values from cancer specific Cox-ph models (when reported in context of all 32 cancer types) were also multiple hypothesis corrected using the FDR method, these corrected p-values were reported as q values for those analyses. The combined effects of the levels of different immune cells on PFS were tested in additive Cox-ph models. The survival analysis for BRCA subtypes was conducted using BRCA subtypes and leukocyte levels as interaction terms in multivariate Cox-ph models. The low iScores for cell types were consistently used as reference groups for all survival analyses, For BRCA subtype analysis low iScores in LumA subtype were used as reference group for all survival analyses. Forest plots were made using the ‘forestplot’ (version 1.7.2) package in R.

### TME map clustering

All cell type iScores were used to create a two-dimensional projection of samples using Tumor Map^70^. We call this projection the Tumor Microenvironment (TME) Map. The spatial coordinates of the TME Map were used to assign the samples into clusters. We used HDBSCAN^71^ clustering method with a minimum cluster size of 20 samples. Survival separation was analyzed between TME Map clusters for each cancer type and each cancer subtype for clusters with at least five samples. Each smaller cluster was compared to the largest cluster for the cancer type or cancer subtype, respectively. Survival was measured in progression free interval, except for Acute Myeloid Leukemia (LAML), which does not have PFI data available and therefore overall survival (OS) data was used for LAML survival analysis. A multivariate Cox proportional hazard (PH) model was used, and the cancer subtype was supplied as a covariate. The p-value for survival separation is measured with a log rank test. For each cancer type and cancer subtype, the difference in each iScore was measured between the largest cluster and each smaller cluster using a t-test. We report the results for the leukocyte iScore as well as the top five most differential iScores ranked by t-test p-value. The deconvolution results for the 9892 TCGA samples are publicly available for interactive visualization and exploration at TME map -(https://tumormap.ucsc.edu/?bookmark=be7404d33f2c10bd6d96eeadc1cd58143daefec320befd396b26de8335c40c33).

### Effect of somatic alterations on infiltration analysis

#### Effect of mutation counts on cell type iScores

To study the cancer-specific effects of total mutation load (synonymous and non-synonymous) on different infiltrating cell types, cell type specific iScores were compared between patients of high-mutation group (upper 25%) and low-mutation group (bottom 25%) using either Mann–Whitney test or Welch’s t-test. Bi-clustering was performed using the QUBIC method^72^*.* The effect of MSI score and POLE mutations status on iScores was tested in a linear regression model as represented below.

*Linear regression model developed for each infiltrating cell type*

$${\text{iScore}}_{{\text{k}}} \sim \, \beta_{0} + \, \beta_{{\text{m}}} {\text{M }} + \, \beta_{{\text{p}}} {\text{P }} + \sum \, \beta_{{\text{n}}} {\text{C}}_{{\text{n}}}$$

*where:*

K represents the infiltrating cell type. M represents the MSI status of the patient (0 if not MSI-H, 1 if MSI-H). β_m_ represents the linear coefficient of M. P represents the POLE mutation status of the patient (0 if not mutated, 1 if mutated). β_p_ represents the linear coefficient of P. C_n_ represents the cancer type (C is 1 for the relevant cancer type and 0 for others, n = 1 to 33). β_n_ represents the linear coefficient of cancer type C_n_. MSI score is obtained from MSIsensor^73^ . All associations were deemed significant at q (FDR) < 0.1.

#### Effect of individual driver gene mutations on iScores

To identify associations between infiltrating cell types and somatic mutations in 299 driver genes^15^, linear models were fit for each cancer. A cumulative pan-cancer linear model was fit to identify associations that exist across cancer types. For all linear models, marginal regression coefficients were obtained. Multiple hypothesis test corrections were performed cancer-wise. The association of iScores with specific variants of each significant mutation was tested using a Welch’s t-test that compared the iScores of patients with that specific variant to iScores of patients with no mutation in the gene of interest. All associations were deemed significant at q (FDR) < 0.1.

*Linear regression model for each combination of driver gene and infiltrating cell type*

$${\text{iScore}}_{{\text{k}}} \sim \, \beta_{0} + \, \beta_{{\text{d}}} {\text{D}} + \, \sum \, \beta_{{\text{n}}} {\text{C}}_{{\text{n}}}$$

*where*:

K represents the infiltrating cell type. D represents the mutation status of the driver gene (0 if not mutated, 1 if mutated). β_d_ represents the linear coefficient of M. C_n_ represents cancer type (C is 1 for the relevant cancer type and 0 for others, n = 1 to 33). β_n_ represents the linear coefficient of cancer type C_n_.

*Linear regression model developed for each combination of cancer type, driver gene, and infiltrating cell type*

$${\text{iScore}}_{{\text{k}}} \sim \, \beta_{0} + \, \beta {\text{d}}_{{\text{D}}} + \, \sum \, \beta_{{\text{j}}} {\text{S}}_{{\text{j}}}$$

*where:*

K represents the infiltrating cell type. D represents the mutation status of the driver gene (0 if not mutated, 1 if mutated). β_d_ represents the linear coefficient of M. S_j_ represents cancer subtypes (j = 1 to J, where J is the number of subtypes available for a given cancer type, S is 1 for the relevant subtype and 0 for others). β_j_ represents the linear coefficient of subtype S_j_.

## Supplementary Information

Supplementary Information 1.

Supplementary Information 2.

## Data Availability

RNASeq expression data (FPKMs) is publicly available on the Genomic Data Commons (GDC) Data Portal (https://portal.gdc.cancer.gov) and was downloaded using the GDC Data Transfer Tool. Mutation calls (https://gdc.cancer.gov/about-data/publications/mc3-2017) and Survival data (https://gdc.cancer.gov/about-data/publications/PanCan-Clinical-2018) are also available for download from the GDC. The iScore calculated for this work were deposited to and can be viewed as a tumor map on: https://tumormap.ucsc.edu/?bookmark=be7404d33f2c10bd6d96eeadc1cd58143daefec320befd396b26de8335c40c33.

## Code availability

The code to estimate cell types using sparse group lasso method is available on GitHub: https://github.com/drisso/deconsgl. The code to generate random pseudobulks and to deconvolve using iScore method is made available at: https://github.com/NCICCGPO/Tumor-Deconvolution-and-Immunogenicity-Manuscript. All other custom R code used for statistical analysis reported in the paper using already available R packages can also be made available upon request from authors.
