# Supplementary material for: Pan-cancer immune and stromal deconvolution predicts clinical outcomes and mutation profiles
Source: Sci Rep. 2025 Jul 4;15:23921. doi: 10.1038/s41598-025-09075-y (PMC12227561; doi:10.1038/s41598-025-09075-y)
Supplement: Supplementary file 1 — Supplementary Information 1. [file 41598_2025_9075_MOESM1_ESM.pdf]

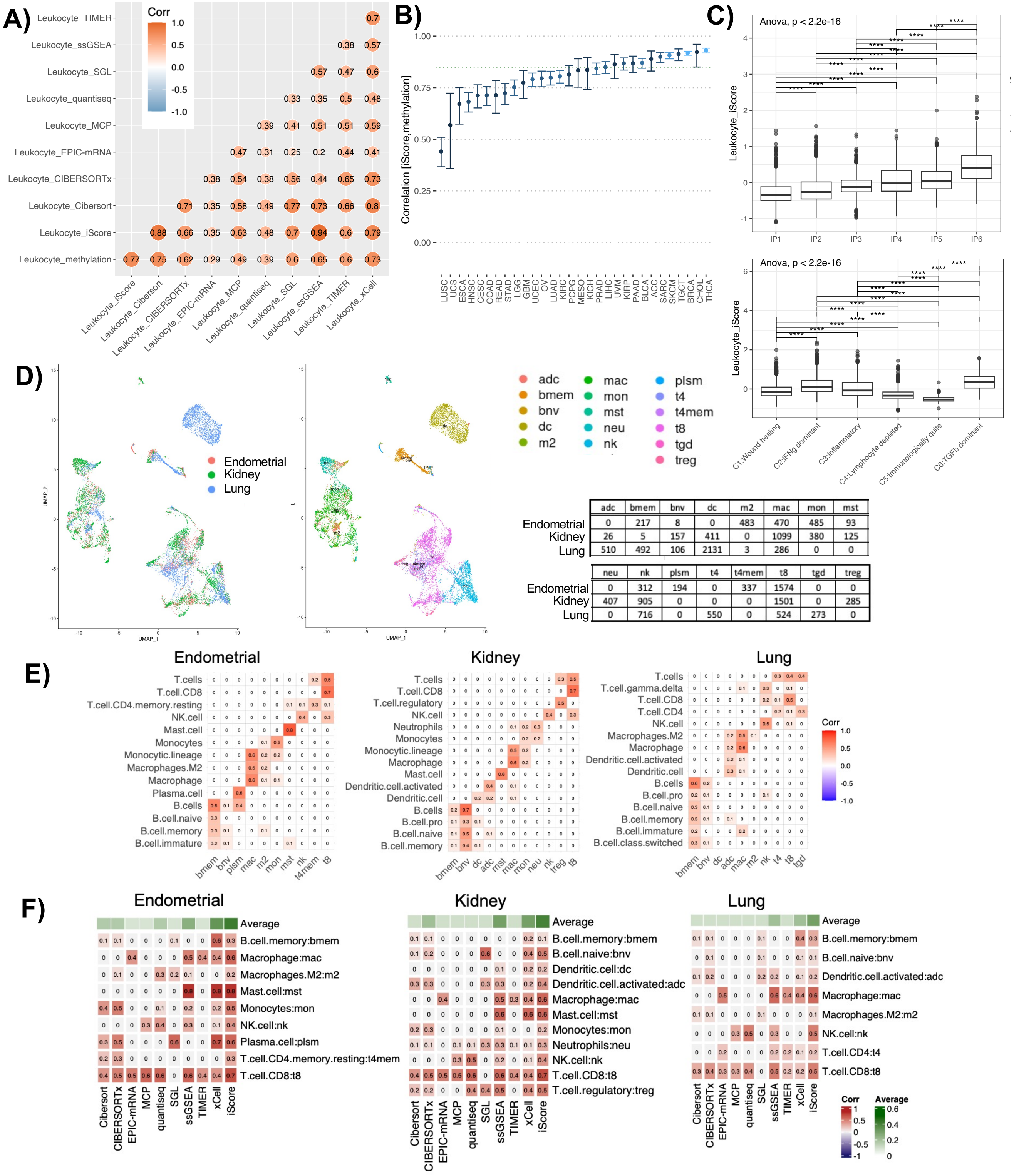

	adc	bmem	bnv	dc	m2	mac	mon	mst
Endometrial	0	217	8	0	483	470	485	93
Kidney	26	5	157	411	0	1099	380	125
Lung	510	492	106	2131	3	286	0	0

	neu	nk	plsm	t4	t4mem	t8	tgd	treg
Endometrial	0	312	194	0	337	1574	0	0
Kidney	407	905	0	0	0	1501	0	285
Lung	0	716	0	550	0	524	273	0

Figure S1: Validation of leukocyte iScores. **A)** Correlations among leukocyte iScore and aggregated leukocyte estimates from individual deconvolution tools. **B)** Cancer-specific correlation coefficients and standard error between leukocyte iScores and DNA methylation leukocyte fractions. **C)** Distribution of leukocyte iScores in the immune subtypes identified in two previously published studies: IP1-6 are immune phenotype clusters from Tamborero et al., where 1 is least cytotoxic and 6 is most cytotoxic immunophenotype; C1-6 are immune subtype clusters from Thorsson et al., (C1: Wound healing, C2: IFNg dominant, C3: Inflammatory, C4: Lymphocyte depleted, C5: Immunologically quite, C6: TGFb dominant) (pairwise $p < 0.05$ adjusted by Bonferroni and marked by asterisk). **D)** scRNA-seq expression profiles from the lung, endometrial, and kidney cohorts projected into the same space in the form of a UMAP. Table shows counts corresponding to the right panel. **E)** Pearson correlations for scRNA-seq based pseudobulks between ground truth (x-axis) and deconvolved cell type iScores (y-axis) using the method described in this paper. Plots show only positive correlations. **F)** Pearson correlations for scRNA-seq based pseudobulks between ground truth (x-axis) and deconvolved cell types using iScores method and the 9 individual tools (y-axis). The white-green scale bar on top shows the average correlation for each tool and iScore across all cell types deconvolved.

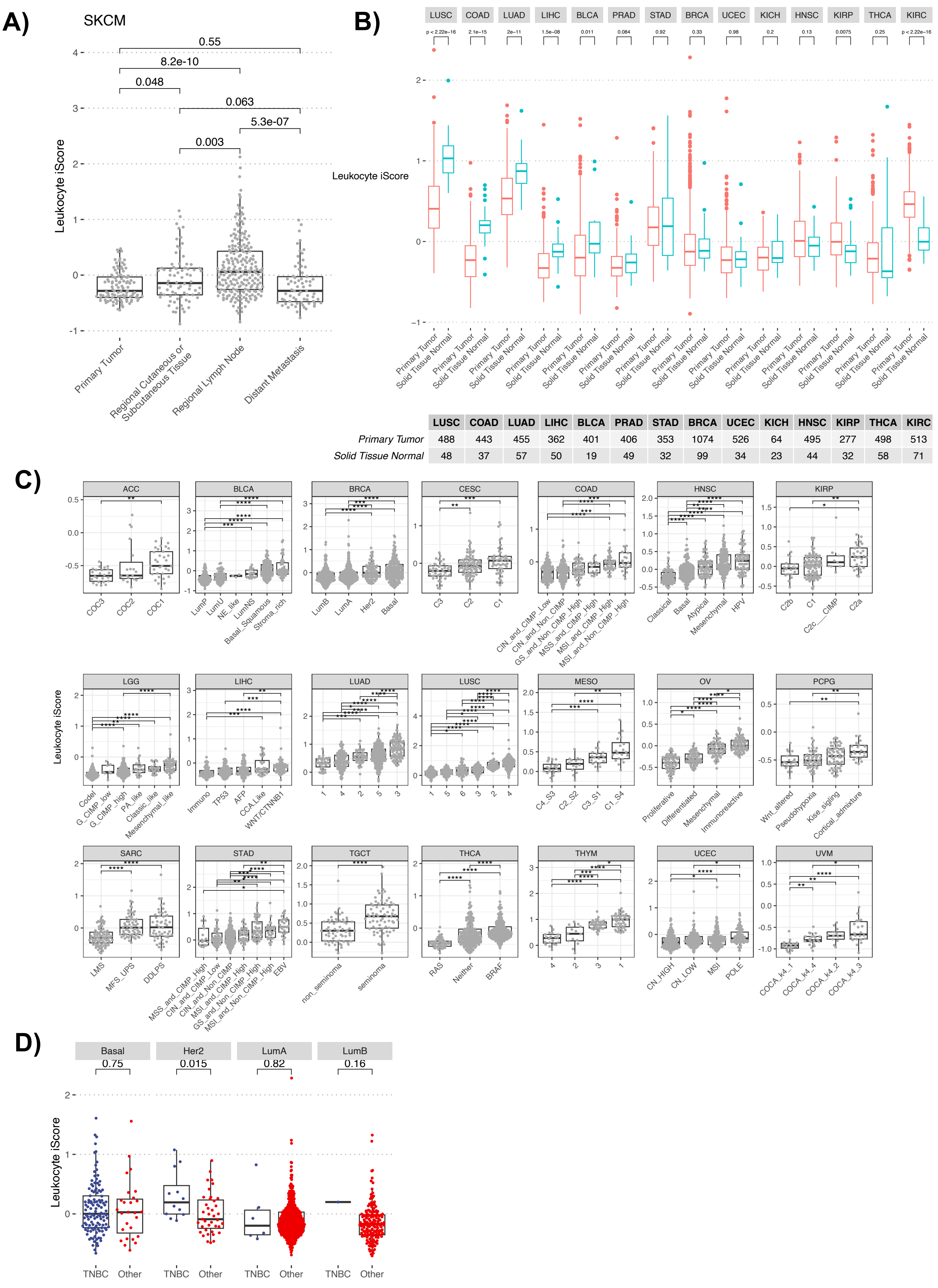

	LUSC	COAD	LUAD	LIHC	BLCA	PRAD	STAD	BRCA	UCEC	KICH	HNSC	KIRP	THCA	KIRC
Primary Tumor	488	443	455	362	401	406	353	1074	526	64	495	277	498	513
Solid Tissue Normal	48	37	57	50	19	49	32	99	34	23	44	32	58	71

Figure S2: Distribution of Leukocyte iScores **A)** in SKCM based on tumor localization, and **B)** between primary and normal tissue samples from select cancer types (minimum n=15 per group), **C)** among cancer subtypes with at least two subtypes showing a significant different in iScore quantification (pairwise $p < 0.05$ adjusted by Bonferroni and marked by asterisk), **D)** between triple negative breast cancer (TNBC) versus non-TNBC (other).

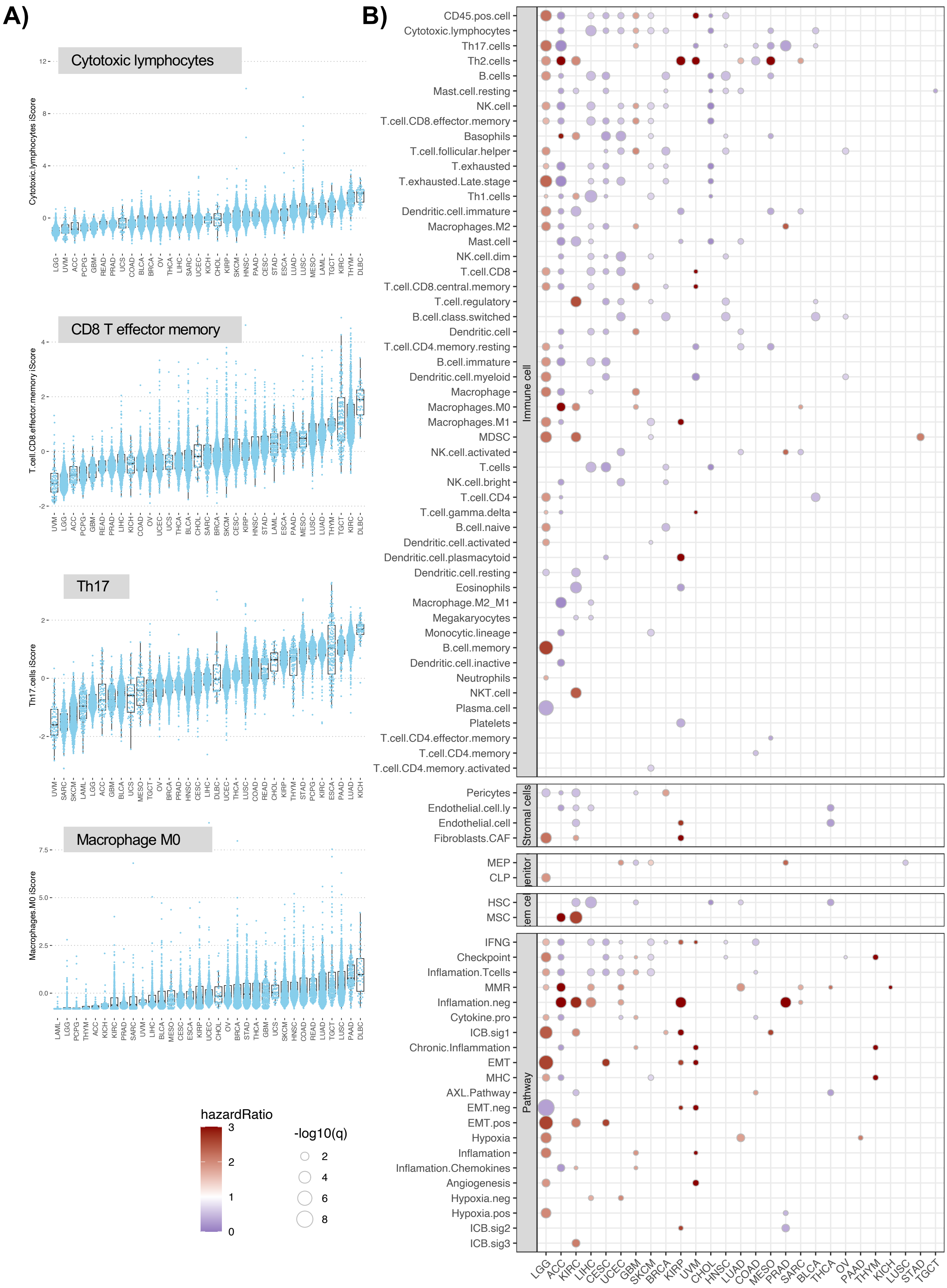

Figure S3: Immune cell type specific iScores and their association with PFS. **A)** Distribution of select cell type iScores ordered by cancer specific medians. **B)** Hazard ratios (HR) for association between PFS and individual cell type iScores (high: top 1/3, low: bottom 1/3, reference group: low). Hazard ratio (HR) and q (FDR corrected log-rank p-value within each cell type) are from multivariate Cox-ph regression models. Visualization is restricted to cancer types significant at q < 0.1.

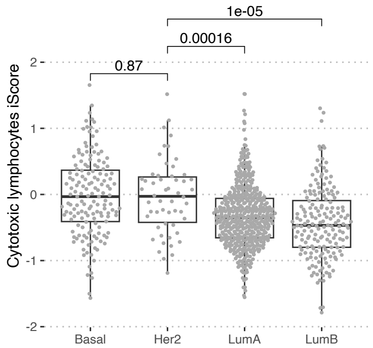

Figure S4: Distribution of cytotoxic lymphocyte (CTL) iScores among BRCA subtypes.

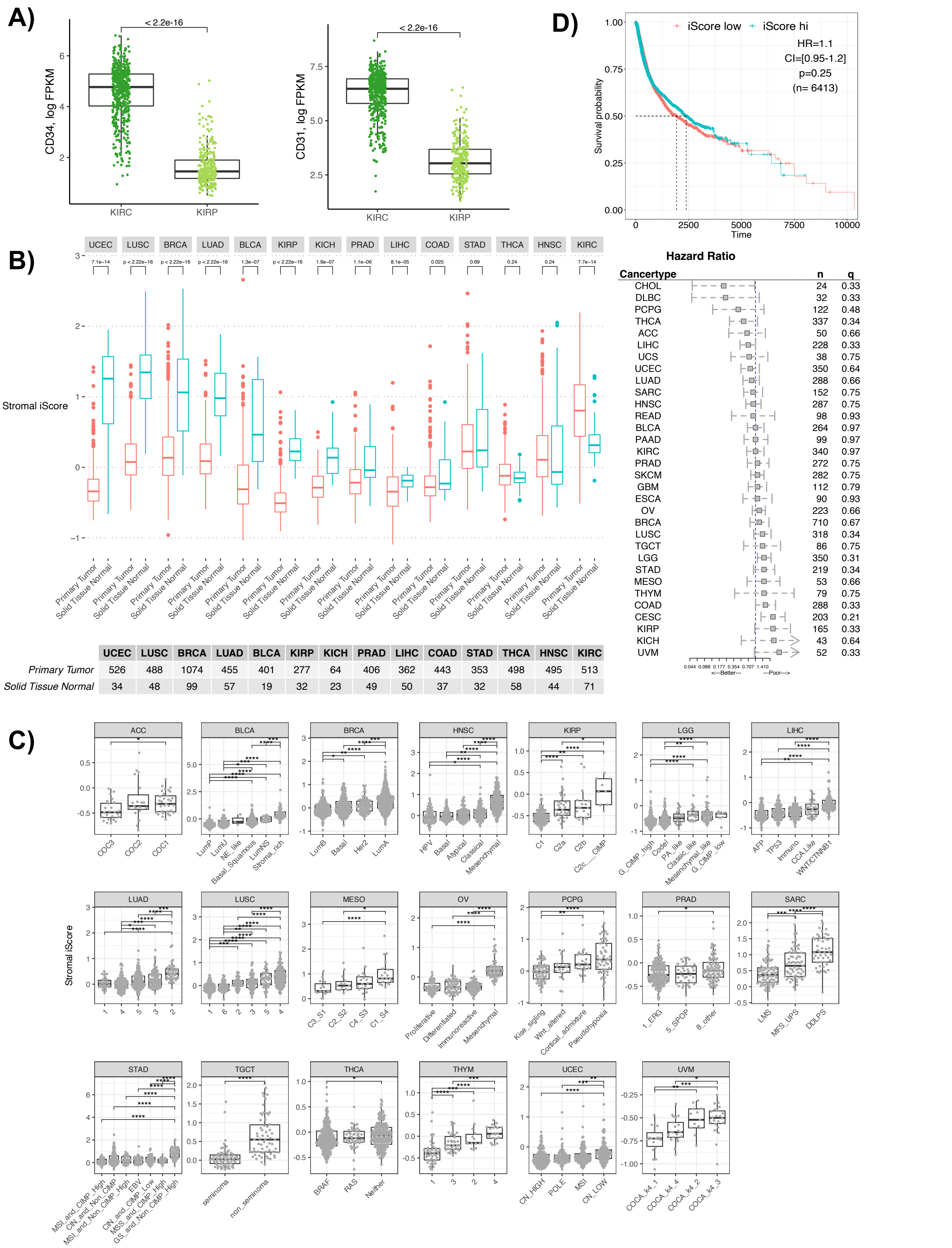

	UCEC	LUSC	BRCA	LUAD	BLCA	KIRP	KICH	PRAD	LIHC	COAD	STAD	THCA	HNSC	KIRC
Primary Tumor	526	488	1074	455	401	277	64	406	362	443	353	498	495	513
Solid Tissue Normal	34	48	99	57	19	32	23	49	50	37	32	58	44	71

Cancertype	n	q
CHOL	24	0.33
DLBC	32	0.33
PCPG	122	0.48
THCA	337	0.34
ACC	50	0.66
LIHC	228	0.33
UCS	38	0.75
UCEC	350	0.64
LUAD	288	0.66
SARC	152	0.75
HNSC	287	0.75
READ	98	0.93
BLCA	264	0.97
PAAD	99	0.97
KIRC	340	0.97
PRAD	272	0.75
SKCM	282	0.75
GBM	112	0.79
ESCA	90	0.93
OV	223	0.66
BRCA	710	0.67
LUSC	318	0.34
TGCT	86	0.75
LGG	350	0.31
STAD	219	0.34
MESO	53	0.66
THYM	79	0.75
COAD	288	0.33
CESC	203	0.21
KIRP	165	0.33
KICH	43	0.64
UVM	52	0.33

Figure S5: Distribution and survival associations for stromal cell types. **A)** Gene expression (quantified as FPKMs) of vascular markers (CD31 and CD34) for renal papillary cell (KIRP) versus renal clear cell (KIRC) cancers. **B)** Cancer specific distribution of stromal iScores between primary and the normal tissue samples (minimum n =15 per group). **C)** Cancer specific distribution of stromal iScores among cancer subtypes for which at least two subtypes show a significant difference (pairwise $p < 0.05$ adjusted by Bonferroni and marked by asterisk). **D)** Pan cancer (top panel) and cancer specific (bottom panel) survival analysis for PFS in patients stratified by individual stromal iScores. Low iScores are reference group Threshold of significance for FDR corrected p-values from multivariate Cox-ph (q) is 0.1.

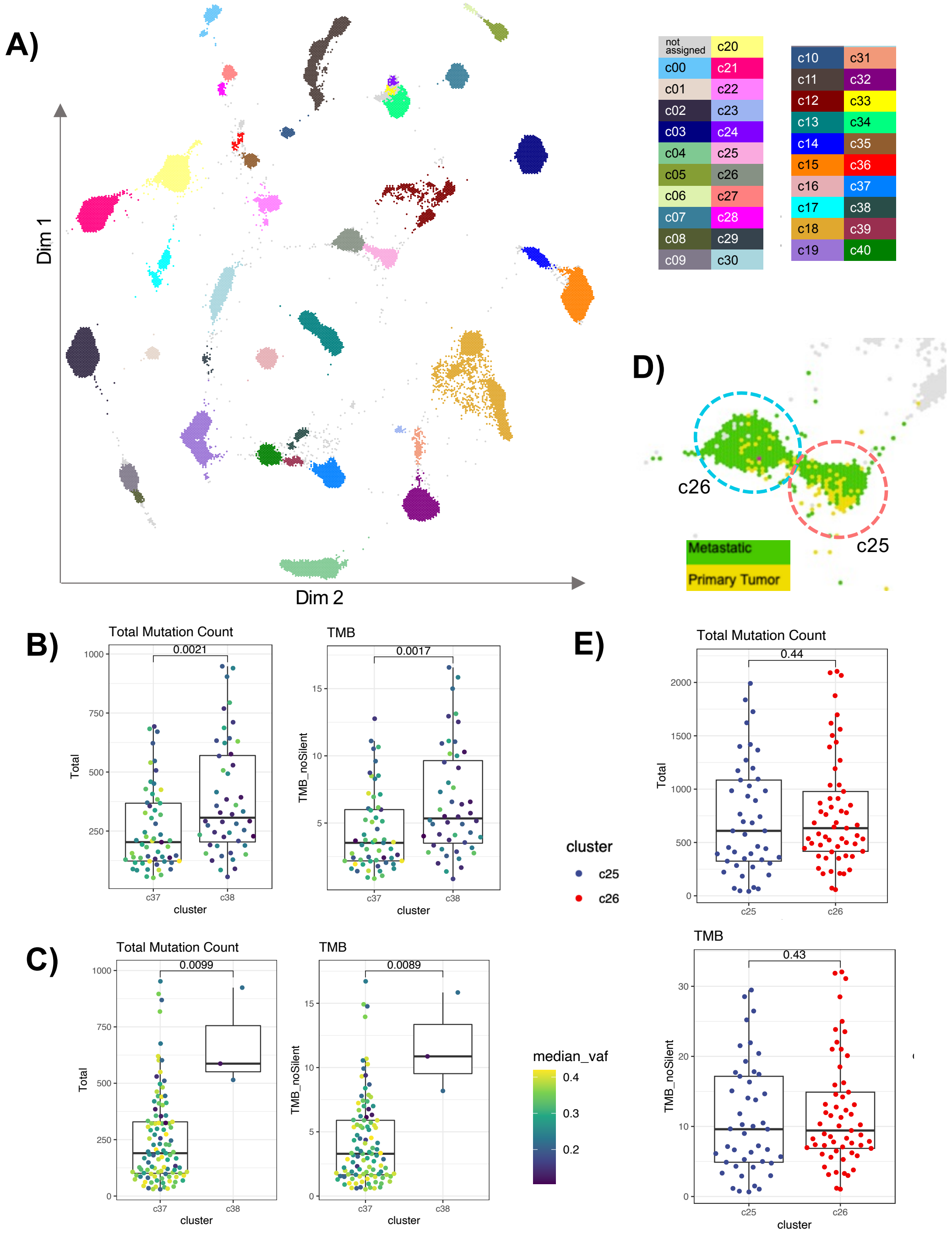

Figure S6: Clustering of the Tumor Microenvironment (TME) map. **A)** Tumor clusters identified using TME map spatial coordinates and HDBSCAN clustering method (n=40). Boxplot showing differences in the total mutation load and TMB between cluster c37 and c38 for **B)** basal squamous and **C)** luminal papillary BLCA subtypes. Points are colored based on the median variant allele frequencies (median VAF) of mutations in each sample. **D)** Primary and metastatic samples in clusters c25 (primary n= 56, metastatic n= 127) and c26 of SKCM (primary n= 18, metastatic n= 198). **E)** Boxplot showing differences in the total mutation load and TMB between cluster c25 and c26 for the RAS hotspot subtype of SKCM.

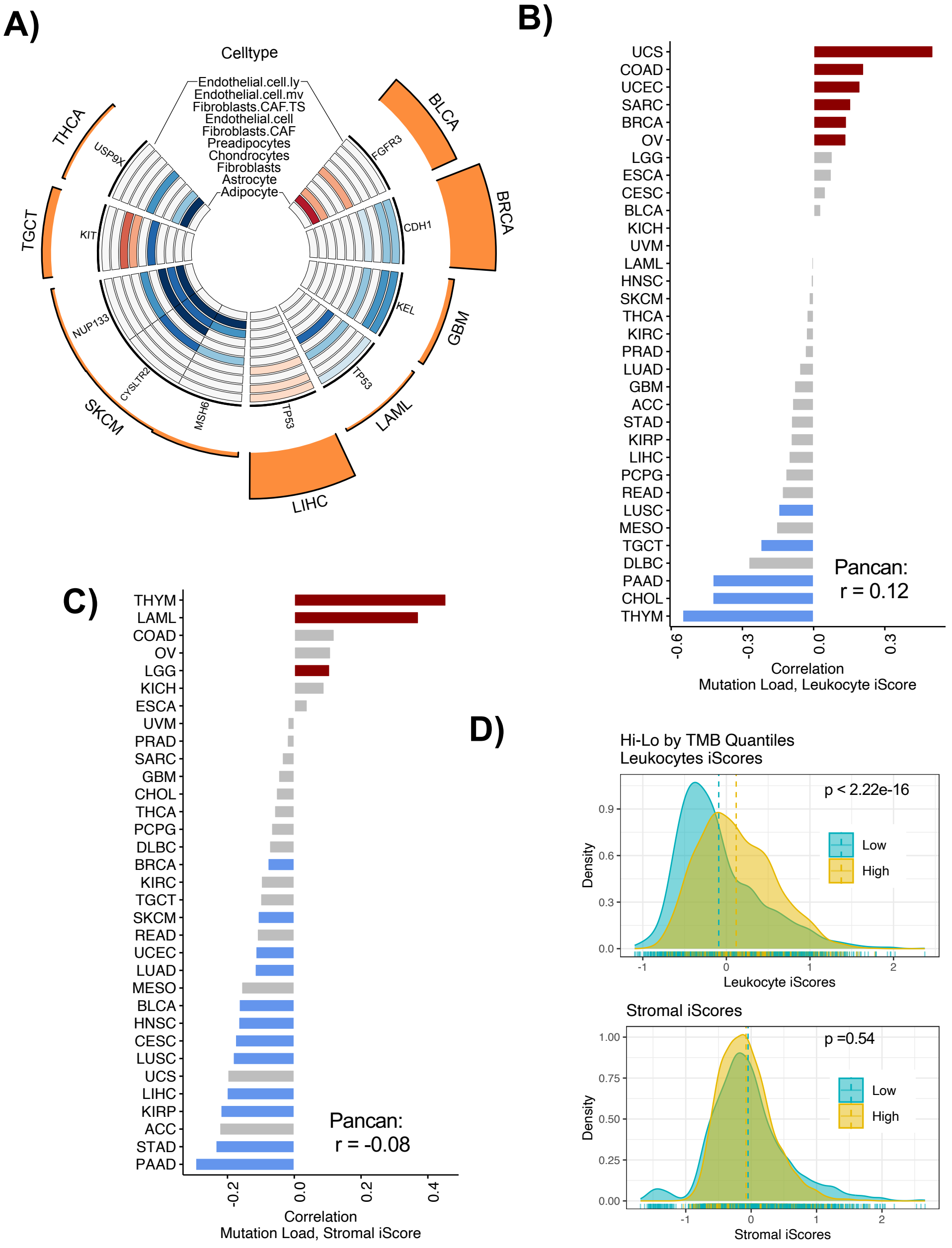

Figure S7: Associations of somatic alterations with cell types**. A)** Circos plot for cancer-specific regression coefficients (coef) in stromal cell types for driver genes mutated in more than 5 tumors per cancer cohort (|coef| > 0.1, p < 0.05). Gene/cell type combinations (n=6) in BRCA CDH1 and SKCM CYSLTR2 with highest statistical significance (FDR < 0.1) are highlighted. Histograms around the circos plot indicate the number of tumors mutated for the corresponding gene. **B)** Cancer specific correlations between TMB and leukocyte iScores. **C)** Cancer specific correlations between TMB and stromal iScore. **D)** Pan-cancer density plots for leukocyte iScores (top panel) and stromal iScores (bottom panel) for tumors segregated by high and low mutation loads, where high is upper 25% and low is bottom 25% of the global mutation load.
